# The effects of modified alternate-day fasting diet on weight loss and CAD risk factors in overweight and obese women

**DOI:** 10.1186/2251-6581-12-4

**Published:** 2013-01-09

**Authors:** Samira Eshghinia, Fatemeh Mohammadzadeh

**Affiliations:** 1 Biochemistry and Metabolic Disorders Research Center, Golestan University of Medical Sciences, Gorgan, Iran; 2 Department of Internal medicine, 5th Azar University Hospital, Golestan University of Medical Sciences, Gorgan, Iran

**Keywords:** Alternate-day fasting, Weight loss, Serum lipids

## Abstract

**Background:**

Obesity is a worldwide health problem with increasing prevalence. Decrease in energy intake has been shown to lower the risk of coronary artery disease in obese subjects. The common form of dietary restriction is daily calorie restriction (CR). Another form is alternate-day fasting (ADF). This study examined the ability of modified ADF to facilitate weight loss and lower cardiovascular risk factors in overweight and obese women.

**Methods:**

15 adult subjects completed an 8 weeks trial (2 weeks observed and 6 weeks ADF). All women consumed very low calorie diet on the fast day and usually diet in every other day. Body weight (BW), fat mass and blood pressure (BP) were measured. Fasting blood samples were collected at the first and 57^th^ day of trial for biochemical analysis.

**Results:**

During the course of the trial, BW of the subjects decreased (p < 0/0001) from 84/3 ± 11/44 kg to 78/3 ± 10/18 kg. Waist Circumference decreased from 87/87 ± 9/74 to 82/86 ± 9/68(p < 0/001). Reduction in systolic BP was seen from 114.8 ± 9.16 to 105.13 ± 10.19 mmHg (p < 0/001) and diastolic BP changed from 82.86 ± 10.6 to 74.5 ± 10.8 (P < 0.05). Total cholesterol decreased from 227/73 ± 49/96 to 214/67 ± 43/27, TG from 160/5 ± 46/18 to 143/9 ± 22/77, LDL from 149/46 ± 49/81 to 131/3 ± 50/97, and FBS from 102 ± 14/7 to 96 ± 11/79 mg/dl but were not significant. HDL increased from 42/32 ± 18/01 to 50/58 ± 19/46 and was not significant.

**Conclusion:**

These finding suggest that short time ADF is a viable dietary option to help obese individuals lose weight and decrease some CAD risk factors. More and longer-term studies in human subjects are needed to support this important result.

## Introduction

Obesity has become an important worldwide health problem, with a rapidly increasing prevalence. The World Health Organization has estimated that by 2015 approximately 2.3 billion adults will be overweight and more than 700 million obese [1]. Obesity is associated with an increased risk of diabetes, cardiovascular events, stroke and cancer [2]. Moreover, a decrease in energy intake has been shown to lower the risk of Cardiovascular Diseases (CVD) such as Coronary Artery Diseases in obese populations [3]. The most common form of dietary restriction implemented is daily calorie restriction (CR), which requires individuals to decrease their energy intake by 15 to 40% of baseline needs - while maintaining adequate nutrient intake each day. Another form of dietary restriction used, although far less commonly, is alternate day fasting (ADF) [4]. The ability of modified alternate day fasting [ADF; i.e., consuming 25% to 40% of energy needs on the fast day and ad libitum food intake on the following day [5] to facilitate weight loss and lower vascular disease risk in human subjects has yet to be investigated. This form of dietary restriction may be more feasible in practice than daily CR. The relation between ADF and cardio protection in human studies remains uncertain. Accordingly, the objective of the present study was to examine the effects of modified ADF diet on body weight, BMI, waist circumference, total body fat mass and cardiovascular risk factors such as systolic and diastolic BP, FBS, serum TG, LDL, HDL and total cholesterol in obese women.

## Material and methods

This study was approved by the research ethics committee of the Golestan University of Medical sciences, Gorgan-Iran. A written consent form was signed by each participant.

### Subjects

Of the 30 overweight or obese women screened, 15 participants completed a 8-wk trial, that includes 2 weeks observed and 6 weeks ADF diet. Baseline characteristics of the participants are shown in Table 1. Inclusion criteria were age 20 – 45 y, BMI ≥ 25 kg/m2, normotensive (<140/90 mm Hg), no weigh changes more and less than 10% for 3 months before the beginning of study, no history of cardiovascular, renal and metabolic disease; nonsmoker. None of the subjects was taking any medication that is known to influence lipid, or glucose metabolism in last 6 months. All women had regularly cycling with menses appearing every 27–32 days. Pregnant women or those trying to become pregnant were excluded. The subjects had similar level of physical activity: less than 3 hours walking in week.

**Table 1:** Baseline characteristics of the participants (n = 15)

Age (y)	33.46 ± 5.9
Weight (kg)	84.3 ± 11.44
BMI (kg/m2)	33.16 ± 5.02
Waist circumference	87.87 ± 9.74
Fat mass (%)	45.82 ± 4.16
Systolic blood pressure (mm Hg)	114.8 ± 9.16
Diastolic blood pressure (mm Hg)	82.86 ± 10.6

All values are presented as Mean ± SD.

### Diet protocol

During the baseline control period (for 2 weeks), each subject was required to keep their body weight stable by maintaining their usual eating and activity habits, then, during 6-week ADF period, all subjects consumed very low calorie diet (25 to 30% energy needs) on the 3 fast day (Saturday, Monday, Wednesday) and then consumed usual diet based on the “Key Recommendations of Dietary Guidelines for Americans” [6] includes about 1700–1800 Kcal/d in every other days (3 days a week). In Friday subjects consume ad libitum without limitation. All foods were prepared in the home and served in 3 meal and 2 or 3 snacks. Energy free beverage (such as water, tea, green tea, coffee without sugar), non starchy vegetable (such as lettuce, cucumber, green leaf, tomato) and sugar free gums were free. Daily dietary carbohydrate, fat and protein accounted for 55, 25, and 20% of ingested energy, respectively.

### Measures

Body weight, BMI, waist circumference, blood pressure and fat mass were measured in first visit and each 2 week throughout period of trial. Body weight was measured with a Seca Digital scale (Clara 803 - Germany) to the nearest 0.1 kg in the evening of fast day, without shoes, and in light clothing. BMI was assessed as the weight in kilograms divided by the square of the height in meters. Fat mass and fat-free mass were assessed using a tetra-polar bioelectrical impedance analyzer (Omron HBF-510). The instrument recorded impedance from hand to foot and consequently, calculated fat mass and skeletal muscle percentage from the impedance value and the personal particulars (weight, height, age, and sex). Waist circumference was measured in triplicate in standing subjects at the midpoint between the lower margin of the last palpable rib and the top of the iliac crest. Blood pressure was measured with the Omron upper arm blood pressure monitor in a seated position after a 10 minutes rest.

Twelve-hour fasting blood samples were collected (at 8.00 – 9.00 am) at the first day of ADF diet and after 6 weeks diet for biochemical analysis. Blood was centrifuged to separate plasma from RBCs, and was stored in freeze (−70°C) until analyzed. Plasma total cholesterol, HDL-C, and triacylglycerol concentrations were measured in duplicate using enzymatic kits, standardized reagents, and standards (Pars Azmoon Co., Tehran, Iran). LDL-C concentration was calculated using the Friedewald equation [7].

#### Statistical analysis

Data were analyzed by using SPSS software (version 16). Results are presented as means ± SEM. Compare between results before and after the trial were assessed by paired *T*-test.

## Results

15 participants completed an 8-wk trial. Baseline characteristics of the participants are shown in Table 1.

During the baseline control period (2 wk) body weight of the subjects was not changed. After the course of the alternate-day fasting diet (6 wk), body weight decreased (P < 0.0001) from mean 84.3 ± 11.44 kg to 78.3 ± 10.18 kg. Mean BMI of the subjects at baseline was 33.16 ± 5.02 kg/m^2^. At the end of the modified ADF course, BMI decreased (P < 0.0001) to 30.72 ± 4.62 kg/m^2^, waist circumference from 87.87 ± 9.74 cm to 82.86 ± 9.68 cm (P < 0. 001). Fat mass was significantly reduced from baseline in all of the subjects. Systolic blood pressure decreased (P < 0.002) from 114.8 ± 9.16 mm Hg to 105.13 ± 10.19 mm Hg and diastolic BP(P< 0.02) from 82.86 ± 10.6 mm Hg to 74.5 ± 10.8 mm Hg.

Changes in mean blood lipid and fasting blood sugar concentrations were not significant after 6 weeks of modified ADF. The anthropometric and biochemical changes are presented in Table 2.

**Table 2:** Anthropometric and Biochemical indexes at baseline and at the end of the trial

**Index**	**Before**	**After**	**P value**
Weight (kg)	84.3 ± 11.44	78.3 ± 10.18	P < 0.001
BMI (kg/m2)	33.16 ± 5.02	30.72 ± 4.62	P < 0.001
Waist circumference	87.87 ± 9.74	82.86 ± 9.68	P < 0.001
Fat mass (%)	45.82 ± 4.16	42.98 ± 4.01	P < 0.001
Systolic blood pressure (mm Hg)	114.8 ± 9.16	105.13 ± 10.19	P < 0.001
Diastolic blood pressure (mm Hg)	82.86 ± 10.6	74.5 ± 10.8	P < 0.05
FBS (mg/dl)	102 ± 14.7	96 ± 11.79	NS
Total cholesterol (mg/dl)	227.73 ± 49.96	214.67 ± 43.27	NS
LDL cholesterol (mg/dl)	149.46 ± 49.81	131.3 ± 50.97	NS
HDL cholesterol (mg/dl)	42.33 ± 18.01	50.58 ± 19.46	NS
Triacylglycerols (mg/dl)	160.5 ± 46.18	143.9 ± 22.77	NS

P <0.05 is statistically significant.

## Discussion

This study shows that modified ADF is an effective, short term dietary intervention to help obese individuals lose weight and total body fat mass. Specifically, we show here that an ADF regimen resulted in a mean 7.1% weight loss of subjects from baseline after 6 wk of diet. Although CR is often used to facilitate weight loss [8,9], many obese patients find it difficult to continue because food intake must be limited every day [10,11]. The ADF is a new strategy that is capable for subject of self-selecting foods to continue their individual feed and fast day energy goals with the help of a dietitian for long time.

Our weight loss findings after 6 wk (6 ± 1.2 kg) are comparable to those of Varady et al. (5.6 ± 1 kg) [4] and Eshghinia et al. (4.7 ± 2.5 kg) findings [12]. Some researcher reported less weight loss due to shorter time of trial. Johnson et al. [13] were shown 4 kg weight loss after 4 wk of ADF in overweight individuals.

According to these effects on body weight, ADF may be considered a suitable alternative to CR to help obese individuals lose weight in short period.

Waist circumference is associated with visceral obesity. Elevated intra-abdominal fat area is associated with elevated risk of coronary heart disease and type 2 diabetes [14,15]. The ability of diet to reduce visceral fat mass has a protective role against theses chronic diseases. In our study, after 6 weeks of ADF diet, waist circumference as the visceral fat mass was reduced by 5.7% (p < 0.001) from baseline. In other studies visceral fat mass was reduced by 4–10% from baseline, that positively associated with body weight loss [4,12,16].

Body composition changes only reported in three of the intermittent calorie restriction human trials with different methods [4,17,18]. In our study, percentage body fat mass declined from 45.82 ± 4.16% at baseline to 42.98 ± 4.01% (p< 0.001) after 6 weeks. Varady et al. [4] assessed fat mass with the same method (tetra-polar bioelectrical impedance analyzer) and reported decreasing of fat mass from 45 ± 1.6% to 42.1 ± 2% (P < 0.05) following 8 weeks ADF. Other trials had used dual-energy X-ray absorptiometry [17,18].

The effects of 6 wk diet on blood pressure were also assessed. Systolic and diastolic BP were lowered (p< 0.05) by 9.7% and 8.3% respectively. Varady et al. [4] reported 5.1% reduce in systolic BP after 8 wk intervention, whereas diastolic BP did not change. Eshghinia et al. findings suggested decrease in systolic and diastolic BP following 4 wk ADF intervention in mild to moderate hypertensive women [12]. Results from another human study indicated that after 3 wk of intervention, neither systolic nor diastolic BP changed in subjects [16].

However, animal data indicate that ADF is effective in decreasing fasting glucose and insulin concentrations, Human trials have noted mixed findings with ADF regarding glucoregulatory function [19]. In this study, fasting glucose concentration decreased following 6 weeks ADF diet but was not statistically significant. Eshghinia et al. found that fasting glucose significantly reduces from baseline following 4 wk ADF in obese women [12]. Heilbronn and colleagues [17] noted a decrease in fasting insulin but no difference in fasting glucose after 22 days of ADF. In another study by the same group [20] men on an ADF diet experienced a reduced insulin response to the test meal, but this effect was not observed in women. In addition, Halberg et al. [18] found no change in fasting glucose or insulin concentrations in men following a 14-day ADF trial. Taken together, these findings about blood glucose concentration suggest that men and women may respond differently to ADF. It seems that in future studies should examine causes of the sex-specific differences noted above.

As a means of assessing cardiovascular response to ADF, trials in this area have examined heart rate, blood pressure, circulating lipids, and ischemic injury. In our study serum TG, total cholestrol and LDL concentration were lowered and HDL increased after 6 weeks. It is possible; these effects resulted from the decreases in body weight and fat mass. However these changes were not statistically significant. Heilbron et al. [17] examined the effect of ADF on CVD risk in both sex. When subjects fasted on alternate days for 3 wk, circulating concentrations of HDL cholesterol increased in the women, whereas triacylglycerol concentrations decreased in the men. There is no clear explanation for these sex-based differences.

Other study reported different result. Varady et al. [4] showed 21.2%, 24.8% and 32% reduction in total cholesterol, LDL and TG respectively but no changes in HDL after 8 wk ADF program. In Eshghinia et al. findings, fasting TG was not changed but total cholesterol, LDL and HDL decreased (P < 0.01). This lack of benefit effect of ADF on HDL cholesterol is not surprising because this cardioprotective lipid parameter is generally increase in response to exercise training [16,21].

It should be noted that this trial and many of other ADF trials had not control groups, included limited subjects and less than 8 weeks duration. A randomized controlled trial with larger groups in longer time needs to test these findings. The next step in the ADF field will be to incorporate an exercise program into this diet to test more benefits effects in lipid profile.

## Conclusion

Our study was shown efficacy of 6 weeks ADF diet to improve several key biomarkers of CAD risk, such as obesity, waist circumference, fat mass, systolic and diastolic blood pressure. The ADF method may be able to modify serum lipid profile. These findings suggest that ADF is a viable diet option to help obese individual loss weight and decrease some CAD risk factors.

## Competing interests

The authors declare that they have no competing interests.

## Authors’ contributions

SE, FM carried out this research, participated in the sequence alignment and drafted the manuscript. We read and approved the final version of manuscript.

## References

[B1] Suzuki K Simpson KA Minnion JS Shillito JC Bloom SR The role of gut hormones and the hypothalamus in appetite regulation Endocrine J 2010 57 5 359 372 10.1507/endocrj.K10E-077 20424341

[B2] Lau DC Diabetes and weight management Prim Care Diabetes 2010 4 Suppl 1 S24 S30 20394888 10.1016/S1751-9918(10)60006-X

[B3] Gomes F Telo DF Souza HP Nicolau JC Halpern A Serrano CV Obesity and coronary artery disease: role of vascular inflammation Arq Bras Cardiol 2010 94 2 255 261 273–9, 260–6 20428625 10.1590/s0066-782x2010000200021

[B4] Varady KA Bhutani S Church EC Klempel MC Short-term modified alternate-day fasting: a novel dietary strategy for weight loss and cardioprotection in obese adults Am J Clin Nutr 2009 90 5 1138 1143 10.3945/ajcn.2009.28380 19793855

[B5] Johnson JB Laub DR John S The effect on health of alternate day calorie restriction: eating less and more than needed on alternate days prolongs life Med Hypotheses 2006 67 209 211 10.1016/j.mehy.2006.01.030 16529878

[B6] Reivew of The Dietary Guidelines for Americans 2010 http://www.health.gov/dietaryguidelines/2010

[B7] Friedewald WT Levy RI Fredrickson DS Estimation of the concentration of low-density lipoprotein cholesterol in plasma, without use of the preparative ultracentrifuge Clinical Chemistry 1972 18 6 499 502 4337382

[B8] Chaston TB Dixon JB Factors associated with percent change in visceral versus subcutaneous abdominal fat during weight loss: findings from a systematic review Int J Obes (Lond) 2008 32 619 628 10.1038/sj.ijo.0803761 18180786

[B9] Varady KA Hellerstein MK Alternate-day fasting and chronic disease prevention: a review of human and animal trials Am J Clin Nutr 2007 86 7 13 17616757 10.1093/ajcn/86.1.7

[B10] Malik VS Hu FB Popular weight-loss diets: from evidence to practice Nat Clin Pract Cardiovasc Med 2007 4 34 41 10.1038/ncpcardio0726 17180148

[B11] Das SK Gilhooly CH Golden JK Pittas AG Fuss PJ Cheatham RA Long-term effects of 2 energy-restricted diets differing in glycemic load on dietary adherence, body composition, and metabolism in CALERIE: a 1-y randomized controlled trial Am J Clin Nutr 2007 85 1023 1030 17413101 10.1093/ajcn/85.4.1023

[B12] Eshghinia S Gapparov GM Effect of short-term modified alternate-day fasting on the lipid metabolism in obese women Iranian J Diabetes and Obesity 2011 3 1 1 5

[B13] Johnson JB Summer W Cutler RG Martin B Hyun DH Dixit VD Pearson M Alternate day calorie restriction improves clinical findings and reduces markers of oxidative stress and inflammation in overweight adults with moderate asthma Free Radic Biol Med 2007 42 665 674 10.1016/j.freeradbiomed.2006.12.005 17291990 PMC1859864

[B14] Scaglione R Di Chiara T Cariello T Licata G Visceral obesity and metabolic syndrome: two faces of the same medal? Intern Emerg Med 2010 5 111 119 10.1007/s11739-009-0332-6 19998063

[B15] Garruti G Depalo R Vita MG Adipose tissue, metabolic syndrome and polycystic ovary syndrome: from pathophysiology to treatment Reprod Biomed Online 2009 19 552 563 10.1016/j.rbmo.2009.05.010 19909598

[B16] Varady KA Bhutani S Klempel MC Kroeger CM Comparison of effects of diet versus exercise weight loss regimens on LDL and HDL particle size in obese adults Lipids Health Dis 2011 10 119 10.1186/1476-511X-10-119 21767400 PMC3150311

[B17] Heilbronn LK Smith SR Martin CK Anton SD Ravussin E Alternate-day fasting in nonobese subjects: effects on body weight, body composition, and energy metabolism Am J Clin Nutr 2005 81 69 73 15640462 10.1093/ajcn/81.1.69

[B18] Halberg N Henriksen M Soderhamn N Stallknecht B Ploug T Schjerling P Effect of intermittent fasting and refeeding on insulin action in healthy men J Appl Physiol 2005 99 2128 2136 10.1152/japplphysiol.00683.2005 16051710

[B19] Trepanowski JF Canale RE Marshall KE Kabir MM Bloomer RJ Impact of caloric and dietary restriction regimens on markers of health and longevity in humans and animals: a summary of available findings Nutrition Journal 2011 10 107 10.1186/1475-2891-10-107 21981968 PMC3200169

[B20] Heilbronn LK Civitarese AE Bogacka I Smith SR Hulver M Ravussin E Glucose tolerance and skeletal muscle gene expression in response to alternate day fasting Obes Res 2005 13 574 581 10.1038/oby.2005.61 15833943

[B21] Ellison RC Zhang Y Qureshi MM Knox S Arnett DK Province MA Lifestyle determinants of high-density lipoprotein cholesterol: the National Heart, Lung, and Blood Institute Family Heart Study Am Heart J 2004 147 529 535 10.1016/j.ahj.2003.10.033 14999205

